# 

**Published:** 1790

**Authors:** 


### CATALOGUE OF BOOKS.

1. A Diſſertation on the Influence of the Paſſions upon the Diſorders of the Body. By *William Falconer*, M. D. F. R. S. 8vo. *Dilly*, London, 1788.

2. Journal of a Voyage to New South Wales; with ſixty-five Plates of non-deſcript Animals, Birds, Lizards, Serpents, curious Cones of Trees, and other natural Productions *. By

* In this work we find an account of two new ſpecies of gum, which are ſaid to poſſeſs valuable medicinal properties. One

By *John White*, Efq. Surgeon General to the Settlement. 4to. *Debrett*, London, 1790.

3. The New Family Herbal; or, Domeftic Phyfician: enumerating, with accurate Defcriptions, all the known Vegetables which are any Way remarkable for medical Efficacy; with an Account of their Virtues in the feveral Difeafes incident to the human Frame. Illuftrated with Figures of the moft remarkable Plants, accurately delineated and engraved. By *William Meyrick*, Surgeon. 8vo. Birmingham, 1790.

4. Truth

One of thefe, or the Red Gum, as it is called, is the produce of a very large and lofty tree, defcribed as a fpecies of *Eucalyptus*. On making incifions in the trunk of this tree, large quantities of red refinous juice are obtained, fometimes to the amount of more than fixty gallons from a fingle tree. When this juice is dried, it becomes a very aftringent gum refin, of a red colour, much refembling that known in the fhops by the name of Kino, and for all medicinal purpofes, we are affured, fully as efficacious. Mr. White adminiftered it to a great number of patients in the dyfentery, and in no one inftance found it to fail. This gum refin diffolves almoft entirely in fpirit of wine, to which it gives a blood red tincture. Water diffolves about one-fixth part only, and the watery folution is of a bright red. Both thefe folutions are powerfully aftringent. — The other fpecies is a yellow refin, the properties of which are faid to vie with thofe of the moft fragrant balfams.

The

4. Truth Vindicated; or, The ſpecific Differences of mental Diſeaſes aſcertained. By *William Rowley*, M. D. Member of the Univerſity of Oxford, the Royal College of Phyſicians in London, &c. 8vo. *Wingrave*, London, 1790.

5. Diſſertatio Medica Inauguralis de Hæmorrhœa petechiali; Auctore *Jacobo Barter Makittrick Adair*, Scoto. 8vo. Edin. 1789.

6. Diſſertatio Medica Inauguralis de Rheumatiſmo; Auctore *Samuele Addiſon*, Britanno. 8vo. Edin. 1789.

7. Tentamen Prophylacticum Inaugurale de

The tree that yields it is about the ſize of an Engliſh walnut tree. This reſin, which exudes ſpontaneouſly from the trunk, is fluid at firſt, but, on being inſpiſſated by the ſun, acquires a ſolid form. Burnt on hot coals it emits a ſmell very much reſembling that of a mixture of balſam of tolu and benzoin, and ſomewhat approaching to that of ſtorax. It is perfectly ſoluble in ſpirit of wine, but not in water, nor even in eſſential oil of turpentine, unleſs it be digeſted in a ſtrong heat. With reſpect to its medicinal qualities, Mr. White obſerves, that in many caſes he has found it a good pectoral medicine, and very balſamic. The tree which produces this yellow gum ſeems to be unknown to botaniſts; but Mr. White (as the Editor of the work informs us) has tranſmitted no ſpecimens by which its genus or even claſs can be determined.

tuenda tuenda Nautarum ſanitate; Auctore *Jacobo Armſtrong*, Hiberno. 8vo. Edin. 1789.

8. Diſſertatio Medica Inauguralis de Typho; Auctore *Jacobo Cugnioni*, Anglo. 8vo. Edin. 1789.

9. Tentamen Phyſiologicum Inaugurale de Somno; Auctore *Nathan Drake*, Anglo. 8vo. Edin. 1789.

10. Diſſertatio Medica Inauguralis de Scorbuto; Auctore *Georgio Dunbar*, Scoto-Britanno. 8vo. Edin. 1789.

11. Tentamen Medicum Inaugurale de Pertuſſi; Auctore *Thoma Harding*, Hiberno. 8vo. Edin. 1789.

12. Tentamen Chemicum Inaugurale de natura Chryſtalliſationis; Auctore *Joanne Benj. Jachmann*, Regiomonto-Boruſſo. 8vo. Edin. 1789.

13. Diſſertatio Medica Inauguralis de Diabete; Auctore *Georgio Jeſſop*, Hiberno. 8vo. Edin. 1789.

14. Diſſertatio Medica Inauguralis de Dyſenteria; Auctore *Andrea Mitchell*, Britanno. 8vo. Edin. 1789.

15. Diſſertatio Medica Inauguralis de Hyſteria; Auctore *Joſepho Mitchell*, Anglo. 8vo. Edin. 1789.

16. Diſ-

16. Diſſertatio Medica Inauguralis de Hydrope Anaſarca; Auctore *Montgomery Nixon*, Hiberno. 8vo. Edin. 1789.

17. Diſſertatio Medica Inauguralis de Urinarum ſtatu et ſignificatione in Morbis; Auctore *Cornelio Van Galen*, Sylva-Ducenſi. 4to. Lugdun. Batav. 1788.

18. Diſſertatio Medica Inauguralis de Ventriculo, plurimorum morborum fonte; Auctore *Wilhelmo Lobé*, Amſtelodamenſe. 4to. Lugdun. Batav. 1788.

19. *Petr. Drieſſen* Oratio Inauguralis de Arte Pharmaceutica in magnum patriæ emolumentum ad majus dignitatis faſtigium evehenda. Folio. Groning. 1788.

20. Anatomicæ Diſquiſitiones de Auditu et Olfactu; Auctore *Ant. Scarpa*. Folio. Pavia, 1789. c. Tab. æn. viii.

21. Philyra qua Academiæ Imperialis Naturæ Curioſorum h. t. Præſes D. *Henricus Fridericus Delius*, Sacri Romani Imperii Nobilis, Comes Palatinus Cæſareus, Conſiliarius intimus Aulicus Brandenburgicus, Medicinæ in Academia Friderico Alexandrina Profeſſor primarius, Academiæ Senior, Acad. Scientiar. Monſpeliens. Rhotomag. et Bavar. Sodalis Perilluſtri Academiæ Cæſar. Natur. Cur. Directori cunctiſque tisque Adjunctis et Collegis, viris generosissimis, illustribus, excellentissimis, prænobilissimis, doctissimis, clarissimisque, quibuscumque demum humanissimis lectoribus S. P. D. Atque de nupero et præsenti dictæ Academiæ statu breviter agit. 4to. Erlangæ, 1789.

22. Dissert. Inauguralis Physiologico-medica de Conceptione et Fœcundatione humana; Auctore *Paul. Fried. Herrm. Grasmeyer*, Hamburgense. 8vo. Gottingæ, 1789.

23. Supplementa quædam ad Dissertationem de Conceptione et Fœcundatione humana; Auctore *P. F. H. Grasmeyer*, Med. et Chir. Doct. 8vo. Gottingæ, 1789.

24. Systematum Generaliorum de Morbis Plantarum brevis Dijudicatio; Auctore *Ulrico Jasper Seetzen*, Sophiengroda-Jeverano. 8vo. Gottingæ, 1789.

25. Dissert. Inaug. sistens Spicilegium Observationum circa partes Genitales masculas Avium; Auctore *Godofredo Guilelmo Tannenberg*, Moscovia-Rosso. 4to. Gottingæ, 1789. c. Tab. æneis iii.

26. Floræ Goettingensis Specimen sistens Vegetabilia saxo calcareo propria; Dissertatio Inauguralis Botanica; Auctore *Henrico Friderico Link*, Hildesiensi. 8vo. Goettingæ, 1789.

27. Defcriptio Pleuritidis, Peripneumoniæ, Pleuropneumoniæ et Anginæ, earumque curatio; propofita a *Ferd. Saalmann*, M. D. 4to. Munfter, 1789.

28. Defcriptio Rheumatifmi acuti, et Dilucidatio ducentorum et quinquaginta Aphorifmorum Hippocratis, ad Rheumatifmum tum acutum tum chronicum; item ad phrenitidem, ad pleuritidem, peripneumoniam, pleuropneumoniam et anginam pertinentium, data a *Ferd. Saalmann*, M. D. 4to. Munfter, 1789.

29. L'Art des Accouchemens, propre aux Inftructions elementaires des Eleves en Chirurgie, neceffaire aux Sages-femmes, &c. par M. *Jof. Charles Gilles de la Tourette*, ancien Eleve a l'Ecole pratique de Chirurgie de Paris, Maitre en Chirurgie, et Demonftrateur Royal de l'Art des Accouchemens a Loudun, Prevot en Charge de fa Compagnie. 2 Tomes, 12mo. Paris, 1787.

30. Memoires phyfiologiques & d'Hiftorie naturelle; par M. *Etienne J. P. Houffet*, D. M. de l'Univerfité de Montpellier, de la Société Royale de Medecine de Paris, premier Medecin des Hopitaux d'Auxerre, et de la Generalité de Bourgogne, pour les Epidemies, &c. 2 Tomes, 8vo. Auxerre, 1787.

31. Me-

31. Memoire qui a remporté le Prix au Jugement de l'Academie de Nancy, le 8 Mai, 1789, fur la queftion fuivante: "1°. Affigner "dans la circonftance prefente (*au mois de Janvier*, 1789) quelles font les caufes qui pour-"roient engendrer des maladies; 2°. Deter-"miner quel fera le caractere de ces maladies, "a l'epoque où les vents du midi et du cou-"chant nous rameneront un tems pluvieux, ou "moins froid; 3°. Indiquer les moyens pre-"fervatifs et curatifs de ces maladies." Par M. *Bouffey*, D. M. Medecin Confultant de Monfieur, et Affocié regnicole de la Société Royale de Medecine, à Argentan. 8vo. Paris, 1789.

32. Journal de Geneve. 4to. Geneva, 1789.

In one of the late fheets of this periodical work (for December 5, 1789) we find a letter from Comte Gorani to Mr. Bonnet, of Geneva, dated October 29, 1789, mentioning a difcovery accidentally made in Catania, by the Chevalier Gioeni, Profeffor of Natural Hiftory, relative to the properties of the volatile alkali fluor in fuppreffing hæmorrhages and healing wounds, when applied topically. According to this account trials have been made with it on birds and quadrupeds; and thofe on quadrupeds have been ſucceſsfully repeated at Naples under the inſpection of Doctors Cothunnio, Vairo, and Sementini, appointed for that purpoſe by the King of Naples. Some experiments, aſcertaining its efficacy in ſuppreſſing hæmorrhages, are likewiſe ſaid to have been made with it, on the human body, in one of the hoſpitals at Naples.

33. Annales de Chimie; ou Recueil de Memoires concernant la Chimie et les Arts qui en dependent: par M. M. *de Morveau*, *Lavoiſier*, *Monge*, *Berthollet*, *de Fourcroy*, le Baron de *Dietrich*, *Haſſenfratz*, et *Adet*. Tomes I. II. 8vo. Paris, 1789.

34. Des Convulſions dans l'Enfance, de leurs Cauſes et de leur Traitement; Ouvrage qui a remporté les deux Prix de la Faculté de Medecine de Paris, et du Cercle des Philadelphes du Cap-François; par M. *Baumes*, Docteur en Medecine de la Faculté de Montpellier, Agregé au College de Medecine de Nimes, &c. 8vo. Paris, 1789.

35. De la Bienfaiſance nationale; ſa Neceſſité et ſon Utilité dans l'Adminiſtration des Hopitaux militaires et particuliers; par M. l'Abbé *Deſmonceaux*, Penſionnaire du Roi. 8vo. Paris, 1789.

36. Etrennes

36. Etrennes d'Hygie; ou Recherches medico-phyfiques fur l'Inoculation de plufieurs Maladies, et particulierement celle de la petite Verole, terminées par un Avis aux Meres de Famille fur leurs Filles de quatorze Ans; par M. *Chevillard*, Docteur en Medecine et en Chirurgie de la Faculté de Montpellier. 12mo. Paris, 1789.

37. Cours elementaire de Matiere Medicale, fuivi d'un Precis de Formules; Ouvrage pofthume de M. *Defbois de Rochefort*, Ecuyer, Docteur Regent de la Faculté de Medecine de Paris, Medecin de l'Hopital de la Charité, &c. 2 Tomes. 8vo. Paris, 1789.

38. *Nicolai Jofephi Jacquin* Collectanea ad Botanicam, Chemiam, et Hiftoriam Naturalem Spectantia, cum Figuris. Vol. II. 4to. Vindobonæ, 1788.

In this work we meet with a botanical defcription of the Aftragalus Exfcapus, which we fhall infert here as a proper fupplement to the account we gave, in a former volume *, of the medicinal properties of that plant:

" ASTRAGALUS EXSCAPUS. *Linn. Syft. pag.*

" 685. *Mant. pag.* 275.

* Vol. IX. page 405.

" Aftragalus

" Aſtragalus perennis, ſupinus; foliis et ſili-

" quis hiſpidis. *Buxb. cent.* 3. *p.* 21. *t.* 38.

" *fig.* 2.

" Glaux montanum acaulon. *Bauh. pin.*

" *pag.* 347. *prodr.* 147.

" Glaux lanuginoſa montana acaulos. *Rupp.*

" *jen.* 2. *pag.* 270.

" Sponte creſcit in Hungaria et Thuringia,

" florens Majo et Junio, fructeſcens Julio. Ra-

" dix perennis, longe fuſiformis, ramo uno

" alterove aucta, perpendicularis, foris fuſce-

" ſcens, intus albida cum centro flavente, odo-

" ris nauſeoſi, ſaporis pauci; adulta tripedalis

" et ſuperne plus minus uncialis diametri,

" multiceps. Folia radicalia omnia, numeroſa,

" ſemipedalia, alia procumbentia, alia erecta,

" tota villoſa, pinnata ex foliolis utrinque nu-

" mero vario ad uſque viginti cum impari, ob-

" longo-ovatis, obtuſis, integerrimis, breviſ-

" ſime petiolatis; coſta terete, rubente. Scapi

" axillares, racemoſi, a quinque ad novem

" flores ſuſtinentes et ipſos breviter peduncula-

" tos, erecti, piloſi, ſemunciales. Bracteæ ſu-

" bulatæ, villoſæ. Perianthium villoſum pal-

" let, longiſſimum, ſemiquinquefidum; den-

" tibus ſubulatis, erectis, acutis, ſubæqualibus.

" Corolla flava ſaturate, erecta. Vexillum cu-

" neiforme,

" neiforme, emarginatum, dorſo carinatum,

" patens. Alæ oblongæ, obtuſæ, vexillo bre-

" viores, ungue gracili et longo. Carina alis

" paulò brevior, emarginata, connivendo ſupra

" unguem lanceolata. Filamenta diadelpha.

" Antheræ luteæ. Germen lineari-oblongum,

" compreſſum, dorſo glabrum, cæterum villo-

" ſum. Stylus àpice ad angulum rectum in-

" curvatum. Stigma obtuſum. Legumen

" cymbæforme, ſive ad alteram ſuturam cari-

" natum, ad alteram depreſſum et planum,

" albidum, piloſum, ſtyli parte acuminatum,

" inflatum, biloculare, in ſutura carinata de-

" hiſcens, pendulum. Semina plura, oblon-

" go-reniformia, glabra, cinereo-fuſca. In

" planta culta caules nonnunquam adſunt pe-

" dales et procumbentes."

39. Memoires ſur les Hopitaux de Paris; par M. *Tenon*, Profeſſeur Royal de Pathologie au College de Chirurgie, des Academies Royales des Sciences, de Chirurgie, et de la Société Royale d'Agriculture de Paris, &c. Imprimés par ordre du Roi. Avec Figures en taille douce. 4to. Paris, 1788.

40. Voyage dans les Pyrenées Françoiſes, dirigé principalement vers le Bigorre et les Vallées; ſuivi de quelques Verités nouvelles et et importantes ſur les Eaux de Bareges et de Bagneres. 8vo. Paris, 1789.

41. Diſſertation ſur le pouvoir de l'Imagination des Femmes enceintes, dans laquelle on paſſe ſucceſſivement en revue tous les grands Hommes qui, depuis plus de deux mille Ans, ont admis l'Influence de cette Faculté ſur le Fœtus, et dans laquelle on repond aux Objections de ceux qui combattent cette Opinion; par M. *Benjamin Bablot*, Conſeiller-Medecin ordinaire du Roi, à Chalons ſur Marne. 8vo. Paris, 1788.

42. Vermiſchte Abhandlungen aus der Arzneywiſſenſchaft; *i. e.* Miſcellaneous Medical Eſſays, by *Aug. Gottlieb Veber*, M. D. 8vo. Halle, 1788.

43. Von der Schwarzgallichten Konſtitution; *i. e.* Of the atrabilious Conſtitution, by *F. X. Mezler*, M. D. 8vo. Ulm, 1788.

44. Briefwechſel ueber die Heilkraefte des Thieriſchen Magnetiſmus, &c. *i. e.* Correſpondence relative to the medicinal Effects of Animal Magnetiſm between Dr. Scherb of Biſchoffszell and Dr. Rahn of Zurich. 8vo. Zurich, 1788.

